# A Randomized Controlled Trial on Pranayama and Yoga Nidra for Anxiety and Depression in Patients With Cervical Cancer Undergoing Standard of Care

**DOI:** 10.7759/cureus.55871

**Published:** 2024-03-09

**Authors:** F. J Nuzhath, N. J Patil, S. R Sheela, G. N Manjunath

**Affiliations:** 1 Department of Integrative Medicine, Sri Devaraj Urs Academy of Higher Education and Research, Kolar, IND; 2 Division of Yoga, Centre for Integrative Medicine and Research, Manipal Academy of Higher Education and Research, Manipal Academy of Higher Education, Manipal, IND; 3 Department of Obstetrics and Gynaecology, Sri Devaraj Urs Medical College, Kolar, IND; 4 Department of Radiation Oncology, Sri Devaraj Urs Medical College, Kolar, IND

**Keywords:** yoga nidra, depression, anxiety, cervical cancer, yoga

## Abstract

Introduction

Cervical cancer might intensify the psychological distress among patients with cervical cancer and the distress caused by the diagnosis and treatment. So, depression and anxiety are at higher levels in patients with cervical cancer. Yoga Nidra and Pranayama are thought to reduce the aftereffects of chemotherapy and radiotherapy potentially. So, in this study, we used the techniques of Yoga Nidra and Pranayama to evaluate their effect on patients with cervical cancer undergoing standard care.

Methodology

Seventy women with cervical cancer were randomized into experimental and control groups. The experimental group of patients with cervical cancer received 30 minutes of yoga intervention twice daily five days a week, for six weeks. The control group was given only the standard of care. The outcome measures were assessed using the Hospital Anxiety and Depression Scale (HADS) questionnaire. The assessment was done at baseline, second, fourth, and sixth weeks.

Results

The results of within‑group comparisons in both groups showed that there was a significant improvement in depression and anxiety scores, with *P* ≤ 0.05 being considered statistically significant. Between groups, analysis shows that in the preintervention, there was no difference between the yoga and control group as *P* > 0.05. After the yoga intervention, there was an enhancement in depression and anxiety scores.

Conclusions

The results of the study concluded that the Yoga Nidra and Pranayama module can be given as adjuvant therapy to the standard of care in patients with cervical cancer for treating the disease and treatment-related anxiety and depression.

## Introduction

Cancer is a catastrophic disease evolving disproportionately across the world, which is treated with conventional treatments to a great extent. They play a vital role in providing patients with a better life. Apart from such qualities, they also have some frailties that can be treated with yoga when provided as an adjuvant to the standard of care. Cancer is considered the second cause of death worldwide. It may take first place in 2060. Based on the World Health Organization (WHO) and the American Cancer Society, the risk of developing cancer is 20.2% for overall 0-74 years. Nearly 180 lakh new cases were diagnosed in 2018. The frequency ratio between men and women is >1 for all cancers [1].

The fourth most common type of cancer is cervical cancer in low- and middle-income countries (LMICs). Ninety percent of cervical cancer deaths occurred in 2015. The fatality of cervical cancer is 18 times higher than that in developed countries [2]. In 2018, 569,847 new cases of cancer of the cervix were diagnosed and 311,365 deaths occurred worldwide [3]. Screening programs have reduced the frequency of cervical cancer. Its fatality has reduced by more than half in 30 years [4]. In 2012, it was the second most frequent type of cancer and third most common cause of cancer death, with a lifetime risk of developing cervical cancer in high-income countries was 0.9% and 1.6% in LMICs.

Intermenstrual vaginal bleeding, postmenopausal vaginal bleeding, postcoital vaginal bleeding, offensive vaginal discharge, and pain abdomen are considered clinical manifestations of cervical cancer [5-7]. Speculum examination, vaginal examination, and rectal examination are considered criteria for cervical cancer diagnosis. A biopsy is needed for confirmation of the disease, and for confirmation of the spread of the disease [8]. The primary mode of treatment is surgery and radiotherapy [9], which is supplemented by chemotherapy [10]. Surgical intervention or chemoradiation is used depending on the stage of the cancer. Chemoradiation treatment can lead to a spectrum of adverse effects, including urinary dysfunction [11,12]. The acute or late effects of the treatment may substantially influence the patient's quality of life [13-15].

Upon a cancer diagnosis or during cancer treatment, multiple physical, social, psychological, and existential stressors emerge, leading to chronic stressors for the patient. This chronic stress causes distress that encompasses various psychological responses, including depression, sadness, anxiety, fear, worry, anger, or panic [16]. Cervical cancer is caused by human papillomavirus, and such patients will be negatively marked by society for having many transient sexual relationships, no willingness for protected sex, and unfaithfulness. The patients also express feelings of shame, self-blame, and fear of social exhaustion. This distinctive property of cervical cancer might exasperate the psychological distress among cervical cancer patients and the distress caused by the diagnosis and treatment. So depression and anxiety are at higher levels in cervical cancer patients [17].

Many ailments can be managed by using complementary alternative medicine (CAM). It helps control the physical, psychosocial, and spiritual aspects of care. Yoga is known as a medicine for the mind and body, and it can be recommended for cancer patients to treat the manifestations and aftereffects caused by the disease and its treatment [18]. Yoga Nidra from the Tantra Yoga tradition, is the best technique to promote complete physical, mental, and emotional relaxation [19].

Pranayama involves a controlled breathing process to improve the pulmonary reserve function and for efficient neurological control [20]. Techniques in yoga like Yoga Nidra and Pranayama are thought to reduce the aftereffects of chemotherapy and radiotherapy potentially. Yoga Nidra can help persons to cope with anxiety and depression [21].

So, in this study, we used the techniques of Yoga Nidra and Pranayama to evaluate their effect on cervical cancer patients undergoing standard of care.

## Materials and methods

Design

This study followed a randomized controlled trial (RCT).

Duration of study

The total duration of the study was two years.

Setting

The study was conducted on patients with cervical cancer who visited the Department of Obstetrics and Gynecology at RL Jalappa Hospital and Research Centre in Tamaka, Kolar, Karnataka.

Mode of selection of subjects

Patients with cervical cancer hospitalized for the standard of care were selected based on the inclusion and exclusion criteria and then randomized into the control and experimental groups using a randomizer.org software tool.

Inclusion criteria

The study includes patients with histopathologically confirmed cervical cancer, specifically in stages IB2 to IVA. Eligible individuals fall within the age range of 35 to 80 years.

Exclusion criteria

The study excluded individuals with cervical cancer who had also been diagnosed with HIV.

Patients with musculoskeletal abnormalities were not included in the study.

Individuals with a history of major surgeries in the preceding four months were excluded from participation.

Sample size calculation

The sample size was calculated using nMaster 2.0 software. The sample size for the study was estimated based on the depression measure between the yoga group and the supportive control group [22]. Considering a power of 90% with an alpha error of 5% with equal allocation and effect size of 80%, the estimated sample size per group was 35, expecting a dropout rate of 10% during the study. The sample size for a group was calculated as 35, and the overall sample size was 70.

**Figure 1:**
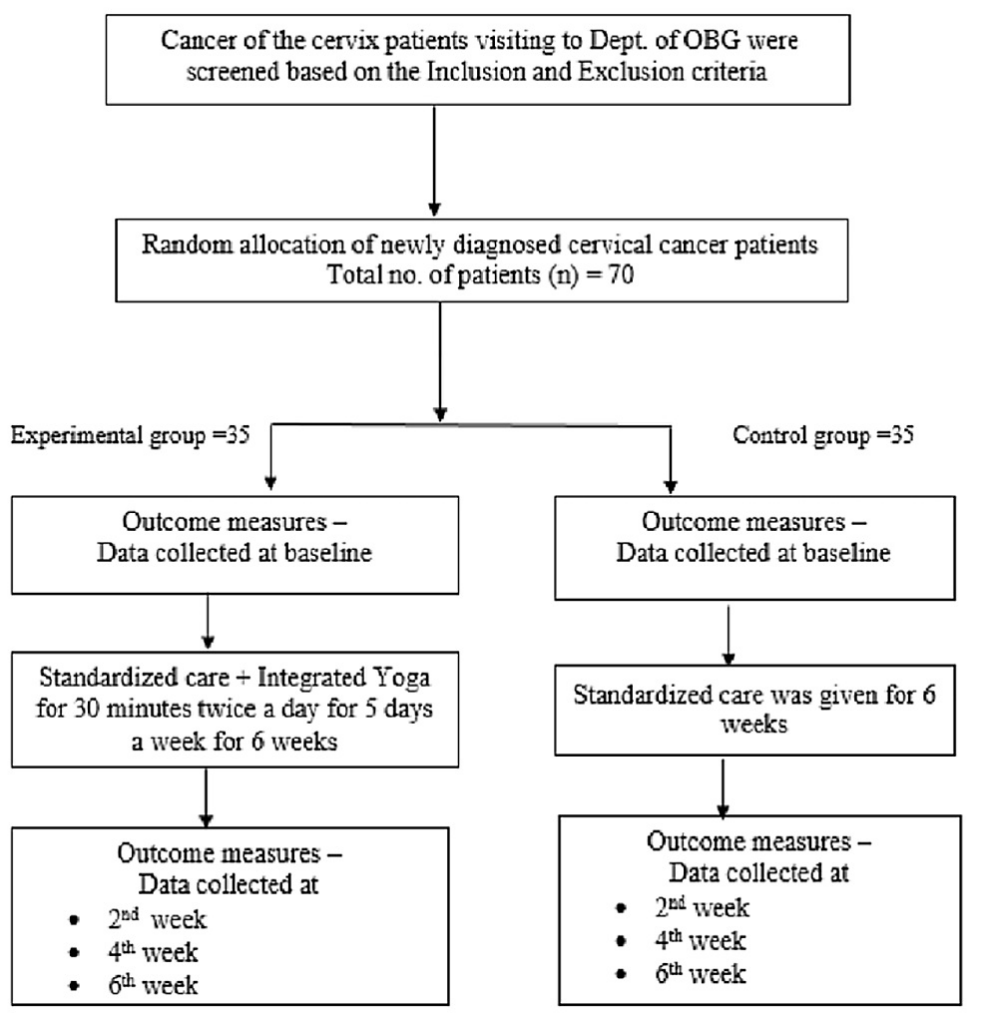
Depiction of the methodological flowchart. Source: [23]. OBG, Obstetrics and Gynecology

The 70 patients were randomly assigned into a control group (*n *= 35) and an experimental group (*n *= 35), as shown in Figure 1 [23].

Procedures

A patient information sheet was given to the patients, and signed informed consent was obtained from all patients who participated in the study. The intervention details were explained explicitly in Kannada and Telugu languages, and all queries were answered before signing the consent form. Patients with cervical cancer who were admitted to the hospital and who fulfilled the inclusion and exclusion criteria were recruited for the study for six weeks. Yoga intervention was given by a yoga professional to the experimental group, and they also received the standard of care. The yoga practice was performed twice daily, five days a week for six weeks. The control group was given a standard of care alone. The standard of care was maintained unaltered throughout the study period for both groups. The study was registered under the Clinical Trials Registry, India, with Registration Number 2022/02/040423.

Yoga Intervention

The integrated yoga module included the practices given in Table 1.

**Table 1:** Integrated yoga module.

SI. no.	Name of the practice	Duration
1	Breathing exercises: hands in and out breathing; hands stretch breathing	5 minutes
2	Pranayama: Nadisodhana Pranayama (alternate nostril breathing); Bhramari Pranayama (bee breathing); abdominal breathing (Deergha Udaraswasan)	15 minutes
3	Relaxation Yoga Nidra	10 minutes
	Total duration	30 minutes

Yoga breathing exercises or Pranayama: Simple management of self-imposed breathing and can be practiced by all age groups and genders. Pranayama is a practice for maintaining physical and mental health disorders related to the mind and body [18]. Yoga Nidra is an organized method to generate material, mental, and emotional relaxation [24].

Outcome measures: The assessment was done by the Hospital Anxiety and Depression Rating Scale (HADS) questionnaire. It consists of 14 questions related to anxiety and depression. Each question offers four choices. Scoring is individually done for anxiety and depression. Based on the scores of anxiety and depression, the patients are categorized as usual, borderline abnormal, and routine.

Data collection and data analysis

Data were collected at the baseline, second, fourth, and sixth weeks. IBM SPSS Statistics for Windows, Version 20.0 (IBM Corp., Armonk, NY) was used for statistical analysis. Repeated analysis of variance (ANOVA) was used to compare the results.

## Results

Skewness in depression and anxiety scores was assessed. As the data exhibited a negative skew, nonparametric tests were employed. The Mann-Whitney U test was used for within-group comparisons, while Friedman ANOVA was employed for between-group comparisons.

Descriptive statistics were conducted for the data, revealing that all study subjects were females from rural areas. Among them, a higher number were found to be illiterate, and they predominantly belonged to a lower economic status, as indicated in Table 2.

**Table 2:** Sociodemographic details of the patients. *n*, number of participants; SD, standard deviation

Sociodemographic variable	All participants (*n *= 70)	Control (*n *= 35)	Experimental (*n *= 35)
Age (years)	Mean ± SD	53.4 ± 12.05	51.1 ± 10.2
Gender	Females (100%)	35 (50%)	35 (50%)
Education	Illiterates (86%)	30 (86%)	30 (86%)
	Literates (14%)	5 (14%)	5 (14%)
Socioeconomic status	Low (93%)	32 (91%)	33 (94%)
	Middle (7%)	3 (9%)	2 (6%)
Religion	Hindu (97%)	35 (100%)	33 (94%)
	Muslim (3%)	0 (0%)	2 (6%)
Marital status	Married (100%)	35 (100%)	35(100%)
Occupation	Agriculture (60%)	21 (60%)	21 (60%)
	Housewives (37%)	13 (37%)	13 (37%)
	Laborer (1.5%)	1 (3%)	0 (0%)
	Vendor (1.5%)	0 (0%)	1 (3%)
Locality	Rural (84%)	30 (86%)	29 (83%)
	Urban (16%)	5 (14%)	6 (17%)

In this study, we compared the depression and anxiety scores of the experimental and control groups at different time points. There was no significant difference in the baseline scores of the experimental and control groups. As the control group was not under any intervention, a significant decline in the mean scores of depression and anxiety scores from week 0 to week 6 in this group can be observed. In the experimental group, as they were on intervention, the mean scores increased over six weeks. The mean scores of depression and anxiety showed significant improvement in the experimental group compared to the control group, as shown in Table 3.

**Table 3:** Hospital Anxiety and Depression Scale (HADS) of control and experimental groups at different treatment time points (between- and within-group analysis). For between-group comparison, we used the Mann-Whitney test, and for within-group comparison, the Friedman-ANOVA test was used. *P *< 0.05 is considered as statistically significant. SD, standard deviation; χ2, chi-square test

	Time points	Control group		Experimental group		Between groups		Within groups			
						*Z*-score	*P*-value	Control group		Experimental group	
		Mean	SD	Mean	SD			*χ*2-value	*P*-value	*χ*2-value	*P*-value
Depression	Week 0	8.4	1.11	7.94	1.16	1.4976	0.1336	88.33	<0.00001	62.99	<0.00001
	Week 2	12.7	1.57	9.5	1.40	6.3194	<0.00001				
	Week 4	14.1	1.23	9.71	1.34	7.07115	<0.00001				
	Week 6	12.2	1.29	7.11	1.75	7.0594	<0.00001				
Anxiety	Week 0	8.2	0.97	8.2	0.95	0.19968	0.84148	70.64	<0.00001	79.99	<0.00001
	Week 2	11.3	1.95	9.88	0.96	3.0951	0.00194				
	Week 4	12.7	1.03	9.74	1.33	6.85972	<0.00001				
	Week 6	11.1	1.29	6.2	1.71	7.14162	<0.00001				

**Figure 2:**
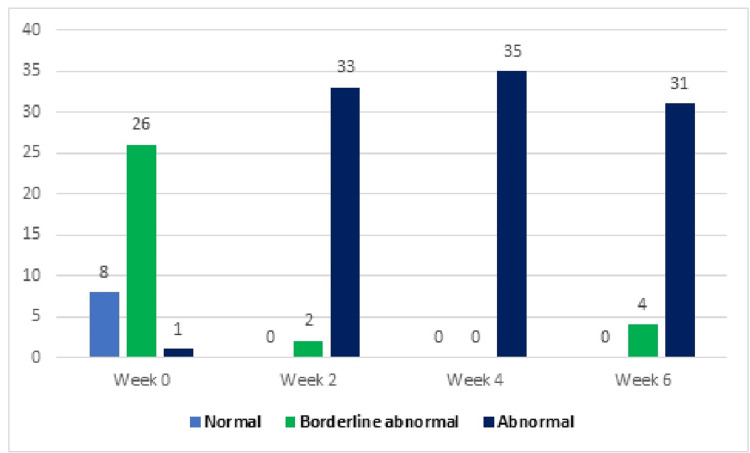
Depression levels in the control group at different time points (n).

**Figure 3:**
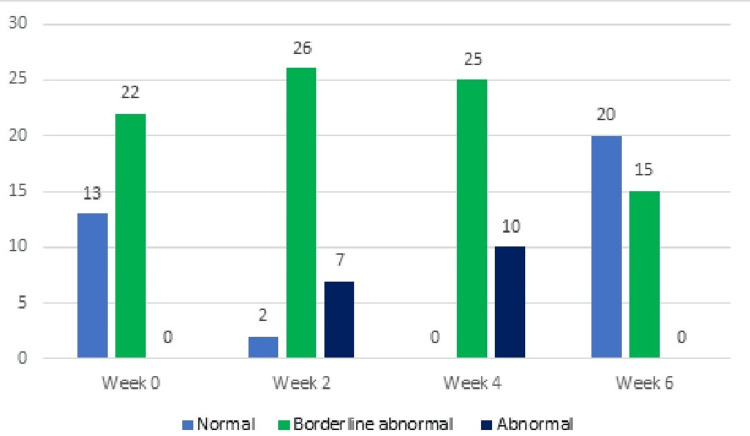
Depression levels in the experimental group at different time points (n).

In the experimental group, subjects dealing with depression from week 0 to week 6 consisted of 13 (37%) categorized as normal, 22 (63%) as borderline, and 0 (0%) as abnormal. However, by the end of week 6, the distribution shifted to 20 (57%) normal, 15 (43%) borderline, and 0 (0%) abnormal cases. In the control group, individuals experiencing depression from week 0 to week 6 included 8 (23%) classified as normal, 26 (74%) as borderline, and 1 (3%) as abnormal. However, by the end of week 6, the distribution shifted to 0 (0%) normal, 4 (11%) borderline, and 31 (88%) abnormal cases, as illustrated in Table 4 and Figures 2-3.

**Table 4:** Depression and anxiety levels of control and experimental groups at different treatment time points.

	Control group (*n* = 35)			Experimental group (*n* = 35)		
Depression	Normal 0-7	Borderline abnormal 8-10	Abnormal 11-21	Normal 0-7	Borderline abnormal 8-10	Abnormal 11-21
Week 0	8 (23%)	26 (74%)	1 (3%)	13 (37%)	22 (63%)	0 (0%)
Week 2	0 (0%)	2 (6%)	33 (94%)	2( 6%)	26 (74%)	7 (20%)
Week 4	0 (0%)	0 (0%)	35 (100%)	0 (0%)	25 (71%)	10 28%)
Week 6	0 (0%)	4 (11%)	31 (88%)	20 (57%)	15 (43%)	0 (0%)
Anxiety						
Week 0	9 (26%)	26 (74%)	0 (0%)	11 (31%)	24 (68%)	0 (0%)
Week 2	0 (0%)	14 (40%)	21 (60%)	0 (0%)	23 (66%)	12 (34%)
Week 4	0 (0%)	0 (0%)	35 (100%)	3 (8%)	18 (51%)	14 40%)
Week 6	0 (0%)	13 (37%)	22 (63%)	27 (77%)	8 (23%)	0 (0%)

**Figure 4:**
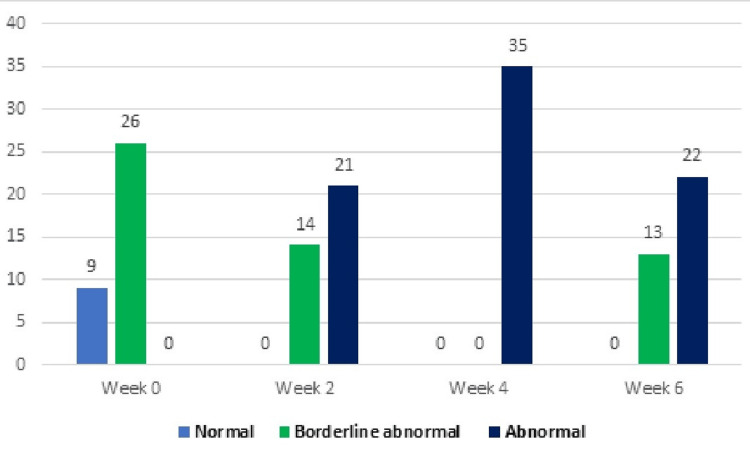
Anxiety levels in the control group at different time points (n).

**Figure 5:**
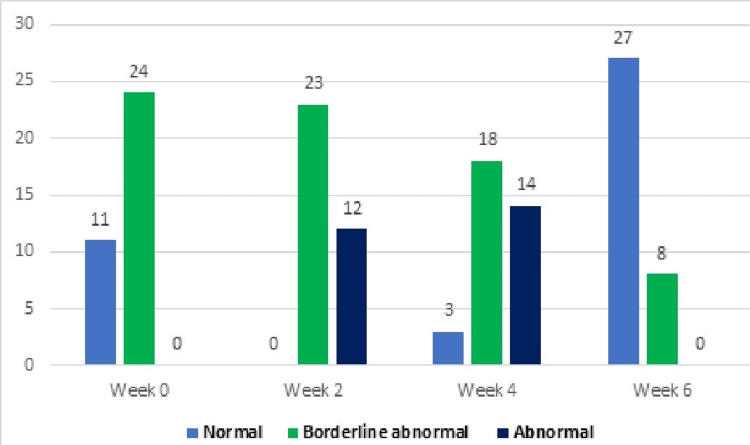
Anxiety levels in the experimental group at different time points (n).

Subjects in the experimental group experiencing anxiety from week 0 to week 6 included 11 (31%) categorized as normal, 24 (68%) as borderline, and 0 (0%) as abnormal. However, by the end of week 6, the distribution shifted to 27 (77%) normal, 8 (23%) borderline, and 0 (0%) abnormal cases. The subjects in the control group dealing with anxiety from week 0 to week 6 are outlined in Table 4, consisting of 9 (26%) normal, 26 (74%) borderline, and 0 (0%) abnormal cases. However, by the end of week 6, the distribution changed to 0 (0%) normal, 13 (37%) borderline, and 22 (63%) abnormal cases, as depicted in Figures 4-5.

## Discussion

In this RCT, scores were improved in both groups. The improvement was highly significant in the experimental group compared to the control group, as the experimental group was undergoing intervention. Compared to baseline, anxiety and depression were reduced considerably after six weeks of yoga intervention. However, anxiety and depression slightly increased during the second and fourth weeks as an adverse effect of the treatment. The increase in depression and anxiety in the control group was higher compared to the experimental group. By the end of the sixth week, anxiety and depression in both groups had reduced, but the reduction was highly significant in the experimental group. The maintenance of the yoga practice appeared 100% by the subjects.

In patients with cervical cancer, the burden on the economy and quality of life is so high and significant that there is a need to prevent and treat this condition [25]. The human papillomavirus significantly impacts the health-related quality of life in emotional [8], social, and sexual functioning [25]. It was seen that higher levels of anxiety, anger, irritability, and depression were noticed in patients with cervical cancer [26]. Depression and anxiety are the usual symptoms of cervical cancer. Patients with cervical cancer need psychological support and the standard of care to cure the disease [27]. Studies have reported a reduction in several cancer-related symptoms by incorporating yoga into the treatment for patients. A study has noted that yoga is beneficial in managing cancer and its treatment-related side effects in patients with breast cancer [22]. A study by Lundt and Jentschke has shown positive changes in anxiety and depression in the long-term post-yoga therapy, and yoga interventions can support cancer treatments [28].

Another study by Rani et al. has explained that psychological problems like anxiety and depression can be improved significantly with Yoga Nidra [29]. Gunjiganvi et al. have determined that Yoga Nidra showed the benefits of improvement in depression and anxiety [30]. Slow Pranayama techniques generate inhibitory signals and hyperpolarizing current within neural and nonneural tissue by mechanically stretching the tissues during breath inhalation and retention. Inhibitory impulses, in cooperation with the hyperpolarization current, initiate the synchronization of neural elements in the central nervous system (CNS), peripheral nervous system, and surrounding tissues, ultimately causing shifts in the autonomic balance toward parasympathetic dominance. From a neuroscientific perspective, the nostrils have a cross-wised interaction with the brain hemispheres [31]. The alteration of nasal airflow through the nostrils modulates contralateral activation of the brain hemispheres, and prolonged expiration, compared to inspiration, may increase parasympathetic activity. Some researchers have found that the long practice of Pranayama also modulates the activity of brain regions involved in emotional processing, such as the amygdala, anterior cingulate, anterior insula, and prefrontal cortex [31]. Neuroimaging studies have shown that Yoga Nidra changes endogenous dopamine release and cerebral flow. Yoga Nidra has an impact on the CNS also. Yoga Nidra has been shown to reduce psychometrically measured indices of mild depression and anxiety [32].

Yoga Nidra and Pranayama are easy and cost-effective practices. During the treatment, patients with cervical cancer have many aftereffects, and these are considered easy and less strained techniques to practice. Patients can engage in Pranayama while in Sukhasana, and practice Yoga Nidra in Shavasana, both of which are relaxed poses. So, these poses themselves are helpful in relaxation, and the yogic practices in these poses are further beneficial in stress reduction, where patients can efficiently perform these techniques. The strengths of the study include the accessibility of yogic relaxation techniques for patients to utilize, coupled with the fact that the intervention was administered twice daily. Inpatients were recruited, making it easy to administer the intervention twice daily without any missed classes or subject dropouts. It's a unique study among cervical cancer studies with a robust methodology.

However, the study had limitations, including its focus on a single hospital, a small sample size, and potential influence on results due to the inclusion of co-morbidities. Therefore, additional studies with larger sample sizes are needed to confirm these findings.

## Conclusions

The study suggests that incorporating a Yoga Nidra and Pranayama module as adjuvant to the standard of care can be beneficial for patients with cervical cancer. This additional approach aims to address not only the physical aspects of the disease but also the mental and emotional well-being of the patients. The study indicates that such a holistic approach may contribute to reducing treatment-related anxiety and depression in patients with cervical cancer. RCTs with higher sample sizes and multicentric studies are advised for future studies for finer results.

## References

[1] Mattiuzzi C, Lippi G (2019). Current cancer epidemiology. J Epidemiol Glob Health.

[2] Cohen PA, Jhingran A, Oaknin A, Denny L (2019). Cervical cancer. Lancet Lond Engl.

[3] Bray F, Ferlay J, Soerjomataram I, Siegel RL, Torre LA, Jemal A (2018). Global cancer statistics 2018: GLOBOCAN estimates of incidence and mortality worldwide for 36 cancers in 185 countries. CA Cancer J Clin.

[4] Rayner M, Welp A, Stoler MH, Cantrell LA (2023). Cervical cancer screening recommendations: now and for the future. Healthcare (Basel).

[5] Petignat P, Roy M (2007). Diagnosis and management of cervical cancer. BMJ.

[6] Lea JS, Lin KY (2012). Cervical cancer. Obstet Gynecol Clin North Am.

[7] Palmer JE, Gillespie AM (20102022). Diagnosis and management of primary cervical carcinoma. Trends Urol Gynaecol Sex Health.

[8] Khushi Khushi (2023). Shaw Gynaecology Book Pdf. Shaw Gynaecology Book Pdf - Medical.

[9] (2013). Cancer survival and prevalence in Australia: period estimates from 1982 to 2010. Asia Pac J Clin Oncol.

[10] Retz M, Karl A (2018). Bladder cancer: current diagnosis and treatment modalities. Urologe A.

[11] Eifel PJ (2006). Chemoradiotherapy in the treatment of cervical cancer. Semin Radiat Oncol.

[12] Maher EJ, Denton A (2008). Survivorship, late effects and cancer of the cervix. Clin Oncol (R Coll Radiol).

[13] Frumovitz M, Sun CC, Schover LR (2005). Quality of life and sexual functioning in cervical cancer survivors. J Clin Oncol.

[14] Korfage IJ, Essink-Bot ML, Mols F, van de Poll-Franse L, Kruitwagen R, van Ballegooijen M (2009). Health-related quality of life in cervical cancer survivors: a population-based survey. Int J Radiat Oncol Biol Phys.

[15] Barker CL, Routledge JA, Farnell DJ, Swindell R, Davidson SE (2009). The impact of radiotherapy late effects on quality of life in gynaecological cancer patients. Br J Cancer.

[16] Andrykowski MA, Lykins E, Floyd A (2008). Psychological health in cancer survivors. Semin Oncol Nurs.

[17] Yang YL, Liu L, Wang XX, Wang Y, Wang L (2014). Prevalence and associated positive psychological variables of depression and anxiety among Chinese cervical cancer patients: a cross-sectional study. PLoS One.

[18] Duncan MD, Leis A, Taylor-Brown JW (2008). Impact and outcomes of an Iyengar yoga program in a cancer centre. Curr Oncol.

[19] Shalmali Shalmali, Das P, Shetty P (2023). Effect of yoga nidra (psychic sleep) in patients with alcoholic hypertensives - a randomized controlled trial. J Ayurveda Integr Med Sci.

[20] Shukla M, Chauhan D, Raj R (2020). Breathing exercises and pranayamas to decrease perceived exertion during breath-holding while locked-down due to COVID-19 online randomized study. Complement Ther Clin Pract.

[21] Singh V, Krishna NR, Bhutia TN, Singh H (2022232023). Effects of virtual iRest Yoga Nidra programme on depression, anxiety, and stress of sedentary women during the second outbreak of COVID-19. J Posit Sch Psychol.

[22] Vadiraja HS, Rao MR, Nagarathna R (2009). Effects of yoga program on quality of life and affect in early breast cancer patients undergoing adjuvant radiotherapy: a randomized controlled trial. Complement Ther Med.

[23] Cuschieri S (2019). The CONSORT statement. Saudi J Anaesth.

[24] Datta K, Tripathi M, Mallick HN (2017). Yoga Nidra: an innovative approach for management of chronic insomnia- a case report. Sleep Sci Pract.

[25] Herzog TJ, Wright JD (2007). The impact of cervical cancer on quality of life--the components and means for management. Gynecol Oncol.

[26] Fleurence RL, Dixon JM, Milanova TF, Beusterien KM (2007). Review of the economic and quality-of-life burden of cervical human papillomavirus disease. Am J Obstet Gynecol.

[27] Kulhara P, Ayyagari S, Nehra R (1988). Psychological aspects of cancer cervix. Indian J Psychol Med.

[28] Lundt A, Jentschke E (2019). Long-term changes of symptoms of anxiety, depression, and fatigue in cancer patients 6 months after the end of yoga therapy. Integr Cancer Ther.

[29] Rani K, Tiwari S, Singh U, Agrawal G, Ghildiyal A, Srivastava N (2011). Impact of Yoga Nidra on psychological general wellbeing in patients with menstrual irregularities: a randomized controlled trial. Int J Yoga.

[30] Gunjiganvi M, Rai S, Awale R, Mishra P, Gupta D, Gurjar M (2023). Efficacy of yoga nidra on depression, anxiety, and insomnia in frontline COVID-19 healthcare workers: a pilot randomized controlled trial. Int J Yoga Therap.

[31] Campanelli S, Tort AB, Lobão-Soares B (2020). Pranayamas and their neurophysiological effects. Int J Yoga.

[32] Pandi-Perumal SR, Spence DW, Srivastava N (2022). The origin and clinical relevance of yoga nidra. Sleep Vigil.

